# Incipient Balancing Selection through Adaptive Loss of Aquaporins in Natural *Saccharomyces cerevisiae* Populations

**DOI:** 10.1371/journal.pgen.1000893

**Published:** 2010-04-01

**Authors:** Jessica L. Will, Hyun Seok Kim, Jessica Clarke, John C. Painter, Justin C. Fay, Audrey P. Gasch

**Affiliations:** 1 Laboratory of Genetics, University of Wisconsin–Madison, Madison, Wisconsin, United States of America; 2 Department of Genetics, Washington University, St. Louis, Missouri, United States of America; 3 Genome Center of Wisconsin, University of Wisconsin–Madison, Madison, Wisconsin, United States of America; Princeton University, United States of America

## Abstract

A major goal in evolutionary biology is to understand how adaptive evolution has influenced natural variation, but identifying loci subject to positive selection has been a challenge. Here we present the adaptive loss of a pair of paralogous genes in specific *Saccharomyces cerevisiae* subpopulations. We mapped natural variation in freeze-thaw tolerance to two water transporters, *AQY1* and *AQY2*, previously implicated in freeze-thaw survival. However, whereas freeze-thaw–tolerant strains harbor functional aquaporin genes, the set of sensitive strains lost aquaporin function at least 6 independent times. Several genomic signatures at *AQY1* and/or *AQY2* reveal low variation surrounding these loci within strains of the same haplotype, but high variation between strain groups. This is consistent with recent adaptive loss of aquaporins in subgroups of strains, leading to incipient balancing selection. We show that, although aquaporins are critical for surviving freeze-thaw stress, loss of both genes provides a major fitness advantage on high-sugar substrates common to many strains' natural niche. Strikingly, strains with non-functional alleles have also lost the ancestral requirement for aquaporins during spore formation. Thus, the antagonistic effect of aquaporin function—providing an advantage in freeze-thaw tolerance but a fitness defect for growth in high-sugar environments—contributes to the maintenance of both functional and nonfunctional alleles in *S. cerevisiae*. This work also shows that gene loss through multiple missense and nonsense mutations, hallmarks of pseudogenization presumed to emerge after loss of constraint, can arise through positive selection.

## Author Summary

Local adaptation is thought to be a driving force in population differentiation and the formation of new species. Yet, there are few examples of ecologically relevant phenotypes that have been mapped to individual genes, making it difficult to know what drives the evolution of such genes and contributes to the molecular mechanisms underlying divergence. Here, we provide a unique case of local adaptation through multi-gene loss. We mapped the genetic basis for natural variation in yeast freeze-thaw tolerance to two water transporters, *AQY1* and *AQY2*. Although tolerant strains harbor functional alleles of both genes, the set of sensitive strains lost aquaporins at least 6 independent times, through missense mutations and frame-shifting deletions. Genome-wide scans reveal several signatures of recent, partial selective sweeps at the aquaporin loci, indicating positive selection for gene loss. This was likely driven by a major fitness advantage of aquaporin loss when cells grow in high sugar concentrations common to many strains' niche. Surprisingly, strains that lost aquaporins also lost the ancestral requirement for these genes during sexual reproduction. This work provides a compelling example of how gene loss through nonsense mutations, a hallmark of pseudogenization, is caused not by loss of constraint but by positive selection.

## Introduction

Biologists have long sought to understand the process of natural selection and the signatures left behind in extant species. Finding evidence of adaptive evolution has been a holy grail for evolutionary biologists, because it can provide insights into how and why organisms evolve. However, examples of adaptive selection from which to glean insights remain relatively scarce [1]. The recent explosion in the number of genomes available for different organisms provides an exciting opportunity to identify loci with unusual patterns of variation indicative of selection (for example [2]–[7]). However, even for loci with strong signatures of selection, the affected phenotypes are often a complete mystery. In contrast, mapping studies link quantitative trait variation to genomic loci that can then be interrogated for evidence of selection. The challenge in most organisms is identifying responsible SNPs within candidate regions, which are often megabases long and contain hundreds of functional elements, hindering further study [8].

Here, we used the power of yeast genetics and genomics to uncover a unique example of adaptive gene loss, involving multiple paralogous genes and several sequential evolutionary events. We previously surveyed phenotypic variation in *Saccharomycetes* collected from diverse environments and found that relatively few of those strains (12%) could survive freeze-thaw (FT) stress [9]. Many tolerant strains were isolated from oak soil in the Northeastern United States, whereas sensitive strains were typically isolated from warm environments, often from fruit or fermentations. This suggested that FT tolerance has been selected for in strains from cold climates but lost in other isolates. Several genes have been linked to freeze-thaw tolerance in yeast and other organisms, including water transporters. The paralogous yeast aquaporins (AQYs) *AQY1* and *AQY2* were implicated in FT stress by the baking industry, which found that AQY over-expression increases yeast viability in frozen bread dough [10]. Rapid export of water through AQYs is thought to increase FT survival by preventing intracellular shearing due to water crystallization [10],[11]. The paralogs may have arisen in the whole-genome duplication (WGD) event in the *Saccharomyces* lineage [12], since all post-WGD species all have two aquaporins whereas most pre-WGD species have a single ortholog (Dana Wohlbach and A.P.G., unpublished). It has been observed that laboratory and industrial strains as well as several vineyard isolates harbor non-functional alleles of *AQY2*, while several strains harbor a non-functional version of *AQY1* [13]–[16]. However, without population-level analysis or knowledge of potential ecological driving forces, it is difficult to distinguish selection at these loci from neutral gene loss in the progenitor of the related strains. Here, we provide the first evidence of adaptive loss of AQY paralogs in natural populations of *S. cerevisiae*, leading to incipient balancing selection via spatial variation in selective pressures.

## Results

**Figure 1:**
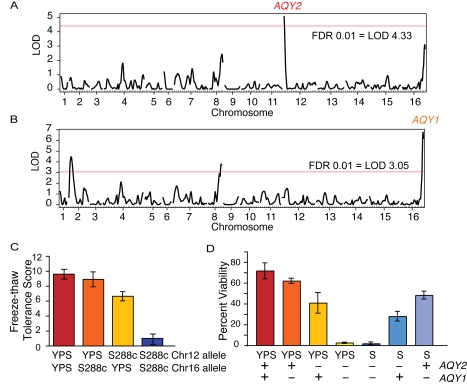
Yeast aquaporins underlie freeze-thaw tolerance in yeast. (A) Genome scan of YPS163 x S288c identified a major QTL on the left arm of Chromosome 12. (B) A second scan in which the first QTL was treated as a fixed term identified a second QTL on the right arm of Chromosome 16. LOD scores at which FDR = 0.01 are indicated by horizontal red lines. Additional peaks in Figure 1B were not significant when both QTL on Chromosomes 12 and 16 were held fixed, suggesting they may be false positives. (C) Effect plot from QTL mapping. (D) Freeze-thaw tolerance was measured in parental strains, YPS163 (‘YPS’) mutants, and S288c-derivative BY4741 (‘S’) harboring empty vector or a plasmid-borne copy of *AQY1* or *AQY2* from YPS163, as described in Methods. The average and standard deviation of biological quadruplicates is shown.

We mapped FT tolerance using a cross between naturally FT-resistant strain YPS163, collected from Pennsylvania oak trees [17], mated to a FT-sensitive lab strain derived from S288c, by phenotyping 44 recombinant strains together genotyped at 198 markers spaced roughly every 60 kb (∼30 cM) [18]. Two loci were identified: one on the left arm of chromosome 12 and one on the right arm of chromosome 16 (Figure 1, see Methods). Each contained one of two paralogous aquaporin transporters, *AQY2* and *AQY1*, respectively, which were previously linked to freeze-thaw tolerance [10],[11],[19]. Together, these genes explained >90% of the phenotypic variation, with *AQY2* alone explaining two-thirds of the effect (Figure 1C). This was confirmed by reciprocal translocation experiments (Figure 1D): deletion of either gene from YPS163 diminished FT tolerance according to the QTL effect plots, while deletion of both genes ablated FT survival. Introducing either gene into the S288c-derived lab strain (which harbors non-functional alleles of both genes [13]–[16]) donated partial FT tolerance to the otherwise sensitive strain. Thus, *AQY2* and, to a lesser extent, *AQY1* are major effectors of natural variation in yeast FT tolerance.

**Figure 2:**
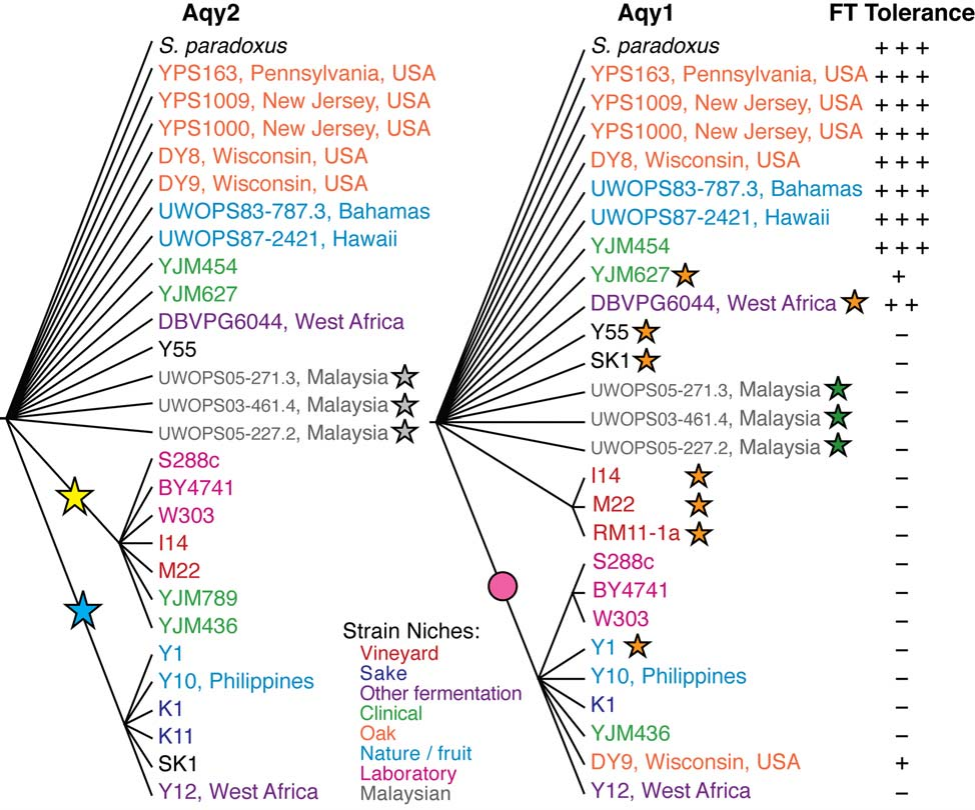
Multiple independent losses of *AQY2* and *AQY1* in diverse *S. cerevisiae* isolates. Bayesian trees of the recapitulated AQY proteins (where gaps were treated as missing data before translation) for Aqy2 (left) and Aqy1 (right). Trees were generated using Mr. Bayes 3.1 and a mixed amino acid replacement model with invgamma rates. All nodes displayed posterior probabilities >0.92. Strains with full-length proteins are nearly identical and do not resolve in the tree. Each star represents the appearance of a different deletion, including the Malaysian G528 (grey), 11-bp (yellow), and Asian G25 (blue) deletions in *AQY2*, and the amino-terminal Malaysian (green) and A881 (orange) deletions in *AQY1*. Appearance of the *aqy1-V121M* polymorphism is highlighted with a pink circle. Strains are color coded according to their niche as shown in the key. Freeze-thaw tolerance scores are listed to the right of strains shown in the Aqy1 tree (with +++ for tolerance and–for sensitivity, see Methods for details).

Sequencing *AQY2* and *AQY1* from the population revealed a near-perfect correlation between FT tolerance and the presence of functional AQY genes. Tolerant strains contained nearly identical and known functional alleles of both genes, while several strains with an intermediate phenotype contained only one functional gene. However, FT-sensitive strains displayed several different non-functional AQY alleles (Figure 2 and Figure S1). There were three distinct frame-shifting deletions in *AQY2*, including a known 11-bp deletion in laboratory and vineyard strains [14],[16], deletion of G at position 25 (G25) in Asian isolates and several other strains [14],[16], and a G528 deletion in the Malaysian *AQY2* that is unable to contribute FT tolerance in our assay (Table S2). Several coding polymorphisms were shared in the recapitulated proteins encoded by the 11 bp-deletion allele or by the G25 allele (Figure 2). There were also three different non-functional *AQY1* alleles in the population, including the A881 deletion that renders *AQY1* inactive in our context (see Table S2 and Figure S5C), the V121M polymorphism known to inactivate water transport [13], and a 955-bp deletion that removes the first 106 bp of *AQY1* and its upstream region in Malaysian strains. The trees for Aqy2 and Aqy1 are distinct from one another, and significantly different from trees based on neutral or genomic sequence that show clear distinction between Asian strains and vineyard isolates [9],[20],[21]. Such discordance between gene and species trees can be a sign of non-neutral evolution. Furthermore, there were five different combinations of non-functional *AQY1* and *AQY2* alleles, and a higher-than-expected frequency of strains harboring both functional or both non-functional genes (p = 3.5×10^−4^, Chi-square test). This cannot be simply explained by shared ancestry, which would have produced similar protein trees and a limited combination of alleles, and instead supports the non-random retention or loss of both AQY genes.

### Signatures of selection at *AQY2* and *AQY1*

We applied several tests to assess if loss of AQY function may have been selected for in some strains. Under the neutral model, the rate of polymorphism within strains should be similar to the rate of divergence across species. Instead, both *AQY1* and *AQY2* show an excess of replacement polymorphism, assessed by the McDonald-Kreitman test [22] that compares non-synonymous (A) to synonymous (S) codon changes (Table S3). *AQY1* showed an A/S ratio of polymorphism (5/4 = 1.25) that was significantly higher than that of divergence (11/49 = 0.22, *p* = 0.026, Fisher's exact test). *AQY2* also showed an excess of polymorphic sites (A/S of 8/20 = 0.4) compared to divergent sites (3/40 = 0.075, *p* = 0.019), as well as an excess of deletions (3/20 versus 0/40, *p* = 0.045). *AQY2* (but not *AQY1*) also deviated from neutral evolution at synonymous sites, showing an excess of SNPs compared to 8 intergenic sequences (*p* = 0.028, multi-locus HKA test [23], Table S4 and Table S5). For the most part, the tests were not significant if subgroups of strains, defined by AQY haplotypes in Figure 2 (see Table S1 for details), were considered separately (Table S3 and Table S5). This result indicates that much of the variation is between strain groups.

Excess polymorphism can result from relaxed constraint in the species, or if local adaptation is driving divergence between populations [24],[25]. To distinguish between these models, we used non-imputed genome sequence data of Liti *et al.* [21] to characterize sequence variation flanking the AQY genes. We applied several empirical tests, which can handle the missing data in the low-coverage genomic sequences and are less subject to the unusual features of *S. cerevisiae* populations (including extensive population structure, unknown population dynamics, ambiguous balance between clonal vs. sexual reproduction, and human-associated migration [21], [26]–[29]) that can confound standard tests [2],[30]. To monitor the variation surrounding *AQY2*, we subdivided 21 strains with data at *AQY2* into strains harboring the Asian G25 deletion, the 11 bp deletion, or the full-length *AQY2* (clonal Malaysian strains were not considered, see Methods). We calculated the average pairwise nucleotide differences surrounding AQY loci within and between groups, and then compared this variation to other regions across the genome. We tested for several signatures: a recent selective sweep is predicted to reduce variation flanking the selected allele in the affected population, while balancing selection can increase variation between strain groups [25]. Since much of the genome may be evolving neutrally, loci with extreme values display the strongest evidence for non-neutral evolution.

**Figure 3:**
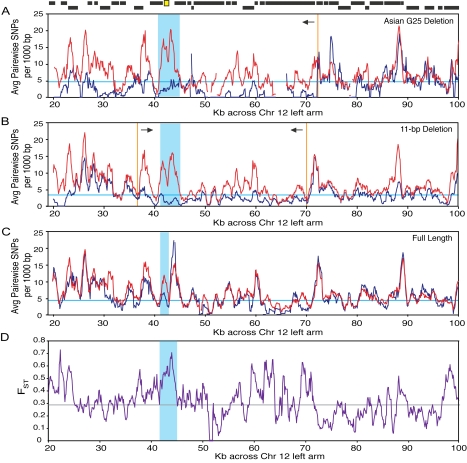
Skewed patterns of variation surrounding the *AQY2* locus. The average number of pairwise nucleotide differences per 1,000 bp sliding window of step size of 100 bp was plotted on Chromosome 12. Variation within (blue curve) and between (red curve) groups of (A) 4 strains harboring the Asian G25 *AQY2* deletion, (B) 16 strains with the 11-bp deletion allele, and (C) 6 strains containing the full-length *AQY2*. Horizontal blue lines represent the genome-wide average of pair-wise variation within each group, and vertical lines represent manually defined breakpoints in trends. Regions identified with skewed between-group minus within-group variation (see Methods) are highlighted in blue. (D) A plot of F_ST_ based on the three groupings in (a–c); horizontal grey line represents the genome-wide average. Gene positions are shown above plots as black boxes, with *AQY2* highlighted in yellow.

For strains harboring the Asian G25 allele of *AQY2*, we saw a high correlation in between-group variation and within-group variation across much of the genome, including the right arm of chromosome 12 (Figure 3A, right side). However, a 50 kb stretch on the left arm of chromosome 12 showed below-average variation within the strains (0.76^th^ percentile compared to other similarly sized regions genome-wide, see Methods) but high variation between groups. There was a sharp break in this pattern at ∼72 kb, which may represent the breakpoint of a selective sweep. To further explore this, we calculated the difference in between-group variation minus within-group variation, then calculated the area under contiguous peaks in the difference curve for comparison (see Figure S2 and Methods). This procedure identified a 5.6 kb region spanning the 870 bp *AQY2* ORF that ranked in the top 1.2 percentile of 4,600 regions genome-wide with skewed between-group versus within-group variation (Figure 3A). Strains harboring the 11-bp deletion displayed a 4,300 bp region encompassing *AQY2* with a significant skew in the between- versus within-group variation (1.8^th^ percentile of 3,238 regions genome-wide, Figure 3B) and below-average within-group variation (6^th^ percentile genome-wide). Strains harboring the full-length *AQY2* showed a smaller peak of 1,800 bp with high between-group variation (6.3^rd^ percentile, Figure 3C), but average within-group variation (>50^th^ percentile). These results show that strains harboring either deletion have low variation within those strain groups, and that the high variation at *AQY2* distinguishes the three groups from one another. Indeed, a genome-wide plot of F_ST_ [2], which measures the population differentiation based on these three groupings, identified a clear peak of 6.4 kb over *AQY2* with above-average F_ST_, ranking among the top 1.5^th^ percentile genome-wide (Figure 3D).

A confounding feature is the extensive population structure within *S. cerevisiae* [21],[29], which can mimic some signatures of selection. Several controls indicate that the observed patterns are unlikely due to demographics. First, these regions were among the most extreme across the genome, which is not expected if population structure is the underlying cause. However, many *S. cerevisiae* strains have mosaic genomes, for which large regions have distinct lineages [21],[29]. To control for this, we performed a partitioning sampling: strains were partitioned at each of 1,370 randomly chosen SNPs across the genome. The difference in between-group minus within-group variation was scored surrounding the partitioning SNP and compared to the difference profile when strains were partitioned based on *AQY2* allele (see Methods). The regions observed for the Asian G25 or 11-bp deletion classes remained among the most extreme (4.7^th^ and 5.4^th^ percentile, respectively). Thus, the profiles we observe in Figure 3A and 3B are uncommonly found at random SNPs, most of which likely reflect neutral variation. In contrast, the skew in variation found in strains with full-length *AQY2* was not significant by this assessment (26^th^ percentile).

We conclude that the observed skew in polymorphism observed in strains with the Asian G25 deletion and the 11-bp deletion in *AQY2* resulted from two separate partial selective sweeps that reduced variation within each group. The high variation distinguishing strain groups is a signature of balancing selection, which may be maintaining both functional and non-functional *AQY2* alleles in the population. Indeed, we observed a positive Tajima's D at *AQY2*, assessed on a smaller set of high-quality sequences (D = 0.851, p<0.05, Figure S3), indicating an excess of intermediate-frequency polymorphism that is consistent with balancing selection [24].

The patterns at *AQY1* were less clear. Strains harboring the *aqy1 V121M* allele or the A881 deletion showed reduced variation within each group and high variation between groups at the *AQY1* region (Figure S4). Although these were highly significant compared to other loci across the genome (0.77^th^ and 1.23^rd^ percentile, respectively), they were not significant compared to random-SNP partitioning described above (16^th^ and 48^th^ percentile, respectively). Thus, the slight skew in between-group versus within-group variation at *AQY1* could be due to demographic factors, incorrect strain groupings, or older or weaker selective sweep(s) that have since recovered variation through recombination or mutation.

### Loss of AQY function provides an advantage in high osmolarity

**Figure 4:**
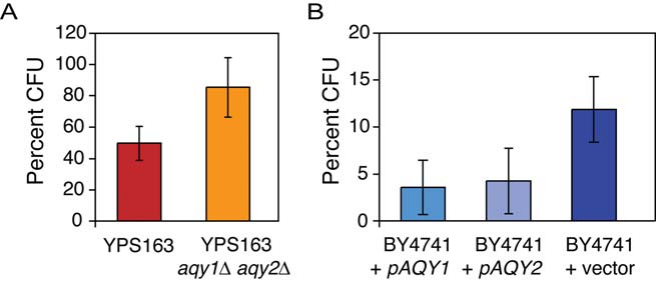
Strains lacking AQY genes show a fitness advantage under high osmolarity. Cells were grown on solid agar plates containing 1.5 M sorbitol for 2 days and the number of colony-forming units (CFU) was compared to a no-stress control plate. (A) CFU for YPS163 and YPS163 *aqy1Δ aqy2Δ* grown on rich medium plus 1.5 M sorbitol; (B) S288c-derivative BY4741 harboring the empty vector or a plasmid expressing the YPS163 allele of *AQY1* or *AQY2*, grown on selective medium with 1.5 M sorbitol. The average and standard deviation of biological triplicates is shown.

The above results strongly suggest selective pressure to lose *AQY* function in some strains, perhaps driven by environmental factors. We previously reported an anti-correlation between FT survival and osmo tolerance across a wide range of *S. cerevisiae* strains (R = −0.35, *p* = 0.006) [9]. Furthermore, a lab strain with functional AQYs was shown to be sensitive to hypo- and hyper-osmotic cycling, but not to consistently high osmolarity [13],[14]. Instead, we found that loss of both AQY genes provides a major growth advantage in high osmolar conditions found in nature (Figure 4). A YPS163 mutant lacking both AQYs displayed ∼1.7X greater survival in 1.5 M sorbitol, whereas introducing a functional AQY into the S288c-derived lab strain decreased survival 2–3X. Furthermore, sorbitol tolerance was anti-correlated to both freeze-thaw tolerance (R = −0.38) and the number of functional aquaporins (R = −0.31) in these strains (Table S1). The sugar concentration used here is comparable to that found in the fruit substrates of many wild strains [31]. Thus, *AQY* function presents a substantial fitness defect in conditions relevant in nature, likely due to passive water loss triggered by the high osmolarity of sugary substrates.

**Figure 5:**
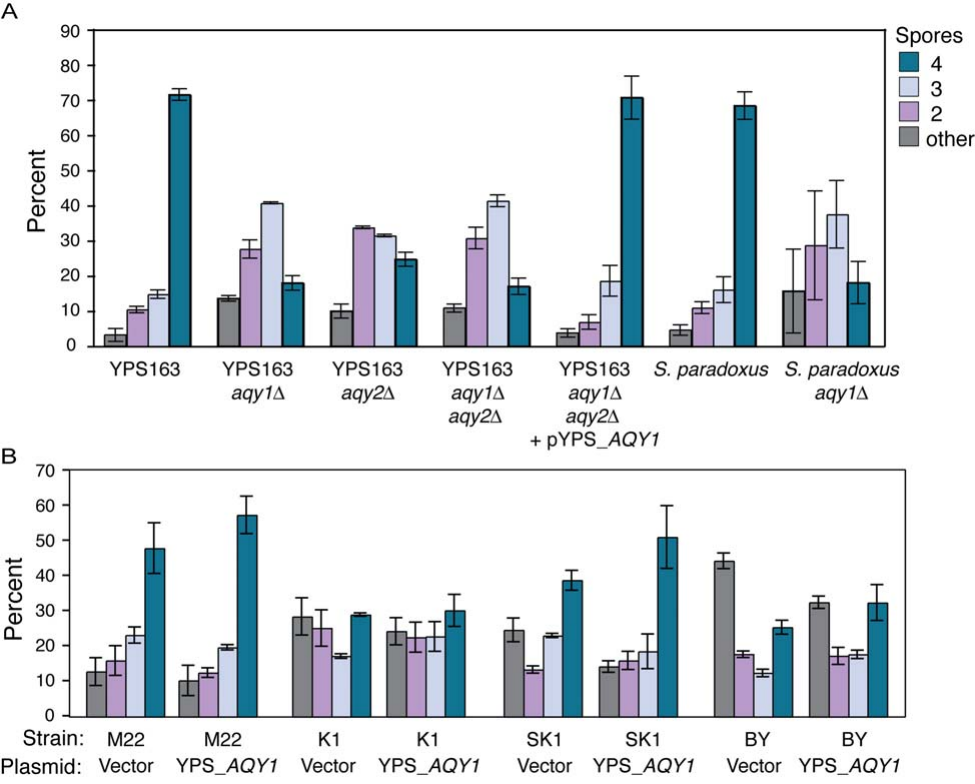
AQY function is required for sporulation in YPS163. (A) Denoted strains were sporulated as described in Methods for 2 days, and the number of events with 4, 3, 2 spores or ‘other’ (representing unsporulated or unscorable cells) was recorded. Each plot represents the average and standard deviation of biological triplicate. Results from *S. paradoxus* strain NRRL Y-17217 are shown here. (B) Vineyard strain M22, sake strain K1, SK1, and S228c derivative BY4743 (BY) harboring empty vector or pYPS_*AQY1* plasmid were sporulated as described for 2–3 d before scoring.

In the course of these experiments, we also discovered that YPS163 lacking either aquaporin had a major defect in spore formation during meiosis (Figure 5). Although *AQY1* had been previously implicated in a late step in spore maturation [32], our phenotype is distinct in that it affects spore production. Whereas >70% of the parental YPS163 formed full tetrads within 2 days, only 18–24% of the double or single mutants produced full tetrads. After 9 days, the mutant produced more spores but was still defective compared to the parental strain (<60% full tetrads compared to ∼85%, Figure S5). The AQY requirement is ancestral, since an *S. paradoxus aqy1Δ* mutant displayed an identical defect (Figure 5A). In contrast, strains without functional AQY genes produce full tetrads (albeit with lower efficiency than YPS163 [33]), consistent with a previous report showing *AQY1* is not required for sporulation in vineyard strains [34]. More importantly, introducing the functional YPS163 allele of *AQY1* into strains with different combinations of non-functional AQY alleles (including strains M22, K1, SK1, and S288c) did not significantly improve spore production (Figure 5B). Thus, strains lacking AQY function have also lost the ancestral need for AQY during spore production.

## Discussion

This work provides the first clear evidence for adaptive loss of AQY function in subgroups of wild *S. cerevisiae* isolates. The excess polymorphism at AQY genes (McDonald-Kreitman and HKA tests), high between-group variation surrounding *AQY2* that distinguishes strain groups (group variation and F_ST_ plots, Figure 3), and skew in the frequency spectrum toward intermediate-frequency *AQY2* alleles (Tajima's D) are all consistent with non-neutral evolution. Furthermore, AQY paralogs have been lost at least 6 independent times, through 2 partial selective sweeps at *AQY2* and possibly others at *AQY1*. The high variation between strain groups, and the non-random retention or loss of both paralogs in diverse strains, is consistent with the establishment of balanced polymorphism. We propose that the antagonistic pleiotropy of aquaporin function, coupled with spatial differences in selective pressures, provide pressure to maintain both functional or both non-functional alleles in distinct subpopulations of *S. cerevisiae*.

FT tolerance may be crucial for survival in cold climates, and along with sporulation efficiency may impart strong pressure to retain AQY genes in strains from wintry niches. Indeed, the ratio of non-synonymous to synonymous differences in YPS163 compared to *S. paradoxus* is 2 - 6X lower for AQYs compared to the genomic average (K_a_/K_s_ of 0.018 and 0.059 for *AQY2* and *AQY1*, respectively, versus 0.1 across all genes [35]). This is consistent with purifying selection acting to remove deleterious codon changes. The oak strains likely represent the ancestral state, since close relatives *S. paradoxus* and *S. mikatae* are also recovered from tree exudates and soil [17],[36], display high FT tolerance [9], and require aquaporins for sporulation (Figure 5 and data not shown). Interestingly, Northeastern-US oak strains display unique phenotypes suggestive of other evolutionary forces as well. *AQY2* is expressed on average 14-fold higher in YPS163 compared to 17 other surveyed strains [9],[37]; those levels are doubled in YPS1009, which underwent a duplication of the entire chromosome 12 [9]. Although further studies will be needed, that over-expression of *AQY2* is known to enhance FT tolerance in industrial strains [10] hints that the elevated expression may have been selected for, further underscoring the importance of AQY function in these strains.

In contrast, many other strains exist in warm environments that never experience freezing. Most of these were sampled from fruit substrates and distillations, which typically consist of ∼25% sugars [31], in contrast to oak soil [38],[39] from which many cold-climate strains have been isolated. Thus, the significant advantage in osmo-tolerance due to AQY loss likely played a major role in selection at this locus. It is unclear which came first–loss of aquaporin requirement during sporulation, or loss of aquaporin function that drove subsequent loss of the sporulation role. Loss of sporulation dependency on aquaporins, coupled with migration to warmer climates, would have relaxed constraint on the genes and facilitated their adaptive loss when cells moved to high-sugar substrates. This model could have involved a single loss of sporulation requirement followed by multiple independent losses of aquaporin function. Alternatively, strong selective pressure to lose aquaporins could have forced multiple independent losses of the sporulation requirement, just as it lead to multiple independent losses of aquaporin function.

*S. cerevisiae* strains are thought to have migrated globally through human association, after two domestication events produced sake/distillation strains and vineyard/wine-making lines ∼10,000 years ago [20],[21],[26],[27],[29],[40]. Human-facilitated migration may have significantly increased exposure of *S. cerevisiae* to diverse climates, which may have imposed new selective pressures when strains encountered new niches. Increased migration may also have facilitated outcrossing of domesticated strains with natural strains, allowing several of these alleles to spread through natural populations. It is important to note that Malaysian strains, not previously associated with domestication events, show unique non-functional AQY alleles, revealing that loss of aquaporins is not strictly driven by domestication.

The selective sweeps of nonfunctional aquaporin alleles appear to have been recent events, given the strength of the signal at *AQY2*, and may reflect an ongoing process. A remaining question is the fate of the emerged balance in polymorphism. Given sufficient migration of strains between the two niches and unequal fitness costs of the opposing haplotypes (i.e. two functional or two nonfunctional AQY alleles), one haplotype may eventually win out to fixation, eliminating the balanced alleles. On the other hand, long-term balancing selection could result if equivalent selective constraints are maintained in each respective niche. In the extreme case, strongly opposing selective forces could restrict yeast migration between environments to promote ecological speciation [41]. Little is known about *S. cerevisiae* migration between tree soil and fruits, although oak-soil strains are genetically well separated from vineyard/fermentation isolates [21],[29],[40],[42]. The antagonistic forces driving aquaporin loss at the cost of freeze-thaw sensitivity may be one factor that has limited gene flow between these niches.

## Methods

### Strains and plasmids

Strains and plasmid constructs are described in Table S6. Two *S. cerevisiae* strains (DY8 and DY9) were isolated from oak-tree soil from Maribel, Wisconsin using the method of [17], and typed by a mating/sporulation assay with a tester *S. cerevisiae* strain (Dan Kvitek and APG, unpublished). Gene deletions were created by homologous recombination, replacing *AQY1* and/or *AQY2* with KanMX3 or NatMX3 drug-resistance cassettes, respectively. Homothalic wild strains capable of mating-type switching (including YPS163, M22, and *S. paradoxus*) were sporulated and dissected, and drug-marked colonies were selected as homozygous diploids. In all cases, homozygous gene deletions were confirmed by diagnostic PCR. The region corresponding to the 870 bp full-length *AQY2* ORF plus 971 bp upstream and 393 bp downstream sequence was cloned from YPS163 or BY4741, by homologous recombination replacing a GFP-*ADH1*-terminator cassette in plasmid BA1924 (provided by P. Kainth and B. Andrews), which is derived from pRS315-based CEN plasmid BA1926 [43] but with the NatMX3 cassette replacing the *LEU2* marker. The region corresponding to the 918 bp full-length *AQY1* ORF with the flanking 947 bp upstream and 747 bp downstream was similarly cloned. All clones were verified by sequencing. To assess functionality of the different alleles, *AQY1* ORFs representing M22, BY4741, or Y55 alleles (identical to the YPS163 allele but harboring the A881 deletion) or the Malaysian *AQY2* allele (identical to YPS163 except for the G528 deletion) were cloned between the native upstream and downstream *AQY1* sequence from YPS163. This was done to prevent confounding influences on expression through variation in the flanking regulatory regions. Plasmids were introduced into YPS163 *aqy1Δ*, BY4741, or other naturally AQY-minus strains, and complementation of spore production in the YPS163 *aqy1Δ* mutant or of FT tolerance in BY4741 was scored (Table S2).

### Phenotyping

Yeast strains were grown at 30°C in YPD medium to an optical density at 600 nm (OD_600_) of 0.3–0.4 in 24-well plates. To measure freeze-thaw tolerance, 200 µl of cells was transferred to 1.5 ml tubes and frozen in a dry ice/ethanol bath (<−50C) for two hours or on ice as control. Viability was measured by scoring serial dilutions spotted onto agar plates as previously described [9], or using Live/Dead stain (Invitrogen, Carlsebad, CA) read on a Guava EasyCyte Plus flow cytometer (Millipore, Billerica, MA). Scores in Figure 2 correspond to high (>80% of YPS163 viability, three pluses), medium (50–80% viability, two pluses), low (<50% viability, one plus), or no detectible (minus sign) FT tolerance. Osmotic tolerance was measured by plating cells onto agar plates containing 1.5 M sorbitol. Percent viability was scored as the number of colony-forming units compared to the no-stress control plate.

### Spore analysis

Cells were grown in YPD rich medium to OD_600 nm_ of 1.0, harvested by centrifugation, resuspended in 1% potassium acetate, and incubated at 25°C for 2 or 9 days. Cells were harvested, diluted and the number of spores per tetrad was counted on a hemocytometer.

### QTL mapping and sequence analysis

QTL mapping strains and analysis were as previously described [18], using the Haley-Knott algorithm implemented in *R-QTL* [44]. Two additional peaks in Figure 1B (left arm of Chromosome 2 and right arm of Chromosome 8) were not significant when Chromosome 12 and 16 QTL were held as fixed terms, suggesting the additional peaks may be false positives. Sequencing using Big-Dye (Applied Biosystems, Carlsbad, CA) scored at least 3 reads (including forward and reverse) per basepair from 2 independent genomic preparations (GenBank accessions GQ848552-74 and GQ870433-54). The vast majority of sequence data represented homozygous sites. The few base pairs with evidence of heterozygocity were represented by one of the alleles, randomly chosen. MK-tests, Tajima's D, and Ka/Ks were calculated in DNASP 5.0 [45] and ML-HKA was done as in [23] using sequence data from [9],[20] and here.

Genome-wide sequence analysis was performed using unimputed, aligned data from [21] with quality scores > = 40 (generously provided by Alan Moses), treating all gaps as missing data to avoid alignment errors. Strains were grouped according to *AQY2* or *AQY1* alleles (see Table S1 for details), and the average number of pairwise SNPs was calculated every 1000 bp with a 100 bp step size, for all pairs of strains within each group and for all strains in a given group compared to each strain outside that group. Within-group variation was scored for all 50 kb regions across the genome with a step size of 20 kb, and for all 5 kb regions with step size of 2 kb. These regions were compared to the 50 kb region highlighted in the text (position 22,000–72,000 in Figure 3) for strains with the G25 *AQY2* allele and to a 5 kb region centered on *AQY2* for other strain groups. All regions were ranked based on the average pairwise within-group variation to calculate the percentile rank of regions in question.

To monitor the skew variation within and between groups, a difference profile of between-group variation minus within-group variation (calculated as described above) was taken across the genome, and all contiguous regions (“peaks”) where the difference value was >1.5X the chromosome-wide average were identified (see Figure S2 and Table S7). The area under each peak was estimated by the trapezoidal method, and compared to the area under the peaks in Figure 3A and 3B spanning *AQY2*. For the partitioning sampling, we scanned for SNPs with at least 3 strains harboring the minor allele, every 10,000 bp across each of the 16 yeast chromosomes. Strains were partitioned based on that SNP, then the between-group and within-group variation was measured for 20,000 bp centered on the partitioning SNP, based on the average-pairwise differences every 1,000 bp with a 100 bp step size as above. A profile of the between-group variation minus the within-group variation was taken in every window. For each partitioning SNP, a peak in the difference profile was identify by walking outward until the difference value was <3.54, the cutoff used the genomic scan shown in Figure 3B. The area under the curve was calculated as above and compared to that measured at *AQY2* by an identical procedure except that strains were partitioned by Asian G25 allele vs. all others strains or by 11-bp deletion vs. all other strains. Very similar percentile rankings were obtained if we scored 5 kb windows centered on each SNP (data not shown).

## Supporting Information

Figure S1 Polymorphisms in *AQY2* and *AQY1*. The plot shows *AQY2* (top) and *AQY1* coding sequences, arranged 5′ (left) to 3′ (right). Blue bars indicate SNPs and orange represents verified gaps in the AQY coding sequences compared to the YPS163 allele for strains in different groups (rows). Strains are organized as shown in Figure 2. Complete sequence data is available through GenBank accession numbers GQ848552-74 and GQ870433-54.
(0.41 MB TIF)

Figure S2 Difference profiles of between-group minus within-group variation. To identify regions with a skew in between-group and within-group variation, we calculated the difference profile as described in the text. Peaks where values were >1.5X the chromosome-wide average were identified and compared to peaks identified at the *AQY2* locus. Blue windows highlight identified peaks over *AQY2*; ORF positions are shown above the figure as described in Figure 3.
(1.54 MB TIF)

Figure S3 Tajima's D at AQY loci. Tajima's D was measured at 12 different loci with high-quality sequence data from [9],[20] and here, in 11 or 12 of 12 strains. Values for the *AQY2* (red) and *AQY1* (orange) coding sequences were compared to other intergenic loci. The 95% confidence interval (mean of non-AQY loci plus two standard deviations) is shown with a horizontal red line. Many genes show negative D values, consistent with previous genome wide estimates for *S. cerevisiae* [29].
(0.22 MB TIF)

Figure S4 Within-group versus between-group variation at *AQY1*. As shown in Figure 3 for strains harboring the V121M allele, A881 deletion, or full-length *AQY1*.
(3.16 MB TIF)

Figure S5 Sporulation defects in mutant strains. (A) Sporulation efficiency as shown in Figure 5 but measured at 9 days. (B) Haploinsufficiency is seen for heterozygous YPS163 *AQY1/aqy1Δ* but not YPS163 *AQY2/aqy2Δ*, suggesting *AQY1* plays a more significant role in YPS163 sporulation. (C) Complementation experiments show that the YPS163 *aqy1Δ* sporulation defect is not complemented by *AQY1* ORFs from S288c derivative BY4741 (BY), M22, or the YPS163 coding sequence with the A881 deletion. To avoid defects due to regulatory differences, each ORF was cloned between the 947-bp upstream and 747-bp downstream sequences from YPS163, exactly as for the pYPS_*AQY1* clone that was able to complement the sporulation defect. Further confirming that these alleles are non-functional in our context, we found that none of these *AQY1* versions contributed FT tolerance to BY4741 (data not shown), unlike the *AQY1* allele from YPS163 (Figure 1C). Although the A881 allele of Aqy1 has been shown to produce a functional water transporter in an *in vitro* system, it was also shown to dramatically reduce protein levels [15], which may explain why it is not relevant in our *in vivo* analysis.
(0.97 MB TIF)

Table S1 Summary of *AQY2* and *AQY1* alleles and phenotypes across strains. The table lists strain names, source of sequences analyzed, allele types, number of functional AQY alleles per strain, and strain phenotypes (as described in Methods).
(0.02 MB XLS)

Table S2 Summary of major-allele phenotying. The table lists the allele tested, functional prediction based on sequence polymorphism, strain from which gene was cloned, and the ability to donate freeze-thaw tolerance to the lab strain or complement the sporulation defect of YPS163 *aqy1Δ*.
(0.02 MB XLS)

Table S3 McDonald-Kreitman tables and p-values. McDonald-Kreitman tests were performed using DNASP on high-quality sequence data from this study, using *S. paradoxus* strain Q69.8, which had the best high-quality sequence coverage [21], as the outgroup. P-values were estimated using Fisher's exact test in DNASP 5.0 [45]. Significant tests are indicated with an asterisk.
(0.06 MB DOC)

Table S4 Input data for ML-HKA test. ^a^ Sample size (number of strains), ^b^ number of segregating sites, ^c^ number of divergent sites. *S. paradoxus* strain Q69.8 was used as the outgroup [21]. We applied the multi-locus HKA method of Wright and Charlesworth [23] to test for selection at *AQY2* and *AQY1*, compared to 7 intergenic sequences with data for both *S. cerevisiae* and *S. paradoxus* [9]. Each fragment is denoted by the chromosome and start position on that chromosome. Due to a large deletion removing the front part of the gene in Malaysian strains, the Malaysian *AQY1* allele started at position 100, removing of an upstream, inframe ATG and 30 additional basepairs that were clearly not orthologous to the full-length *AQY1* from other strains. Intergenic regions were analyzed after removing two clearly non-orthologous regions from all strains (350 bp from the chr2 fragment and 387 bp from the chr16 fragment), the result of apparent recombination in subgroups of strains. This dataset amounted to 183 and 150 silent positions in *AQY2* and *AQY1*, respectively, and 3,337 scorable sites across 7 intergenic fragments.
(0.07 MB DOC)

Table S5 Results of ML-HKA analysis. ML-HKA tests were run under a model in which all loci were evolving neutrally and compared to models in which *AQY2*, *AQY1*, or *AQY2* and *AQY1* were under selection. The program was run with chain length 100,000 in all cases. P-values were calculated based on the chiX distribution of the likelihood statistic (2*(ln L_selection model_–ln L_neutral model_) listed degrees of freedom (df), as described (Wright and Charlesworth 2004). There was no increase in significance when all sites in *AQY2* and *AQY1* were treated as silent (justified since the genes are non-functional) (data not shown). We also ran the analysis separately for only strains in Full-length group, Asian G25 deletion group, or 11 bp-deletion group. Variation at *AQY2* and *AQY1* within each group was compared to variation at other loci only for strains in that group. The results were the same when the genes in question were scored against intergenic sequences from all available strains. None of the tests were significant in any case.
(0.06 MB DOC)

Table S6 Strains and plasmids used in this study.
(0.02 MB XLS)

Table S7 Peak areas identified in *AQY2* scan. Each column shows the chromosome, start position of peak, length of identified peak, maximum difference in between-group variation minus within-group variation, and area under the difference curve.
(0.19 MB XLS)
