# Supplementary figures and images for: Incipient Balancing Selection through Adaptive Loss of Aquaporins in Natural Saccharomyces cerevisiae Populations
Source: PLoS Genet. 2010 Apr 1;6(4):e1000893. doi: 10.1371/journal.pgen.1000893 (PMC2848549; doi:10.1371/journal.pgen.1000893)

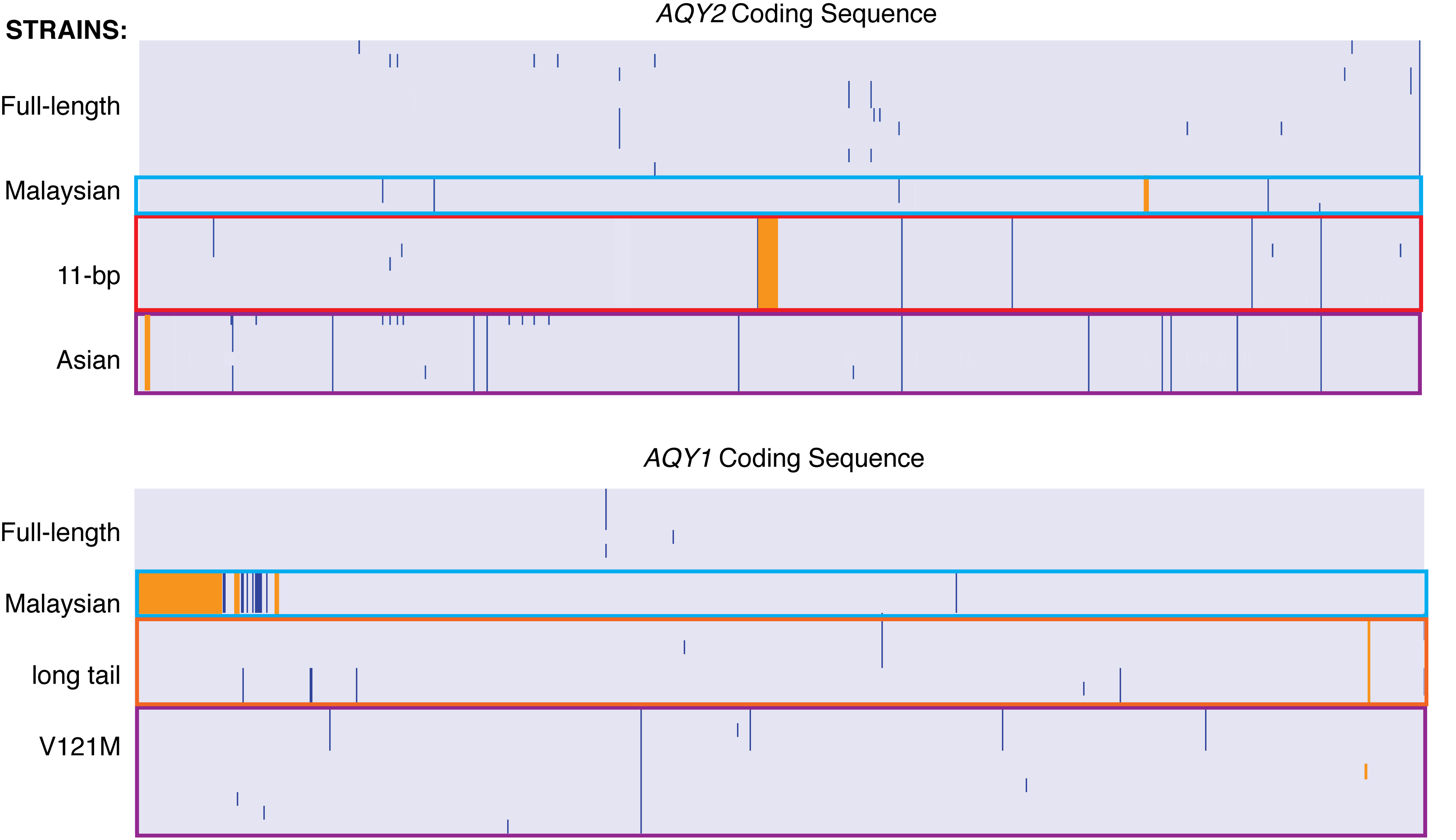

Supplement: Figure S1 — Polymorphisms in AQY2 and AQY1. The plot shows AQY2 (top) and AQY1 coding sequences, arranged 5′ (left) to 3′ (right). Blue bars indicate SNPs and orange represents verified gaps in the AQY coding sequences compared to the YPS163 allele for strains in different groups (rows). Strains are organized as shown in Figure 2. Complete sequence data is available through GenBank accession numbers GQ848552-74 and GQ870433-54. (0.41 MB TIF) [file pgen.1000893.s001.tif]

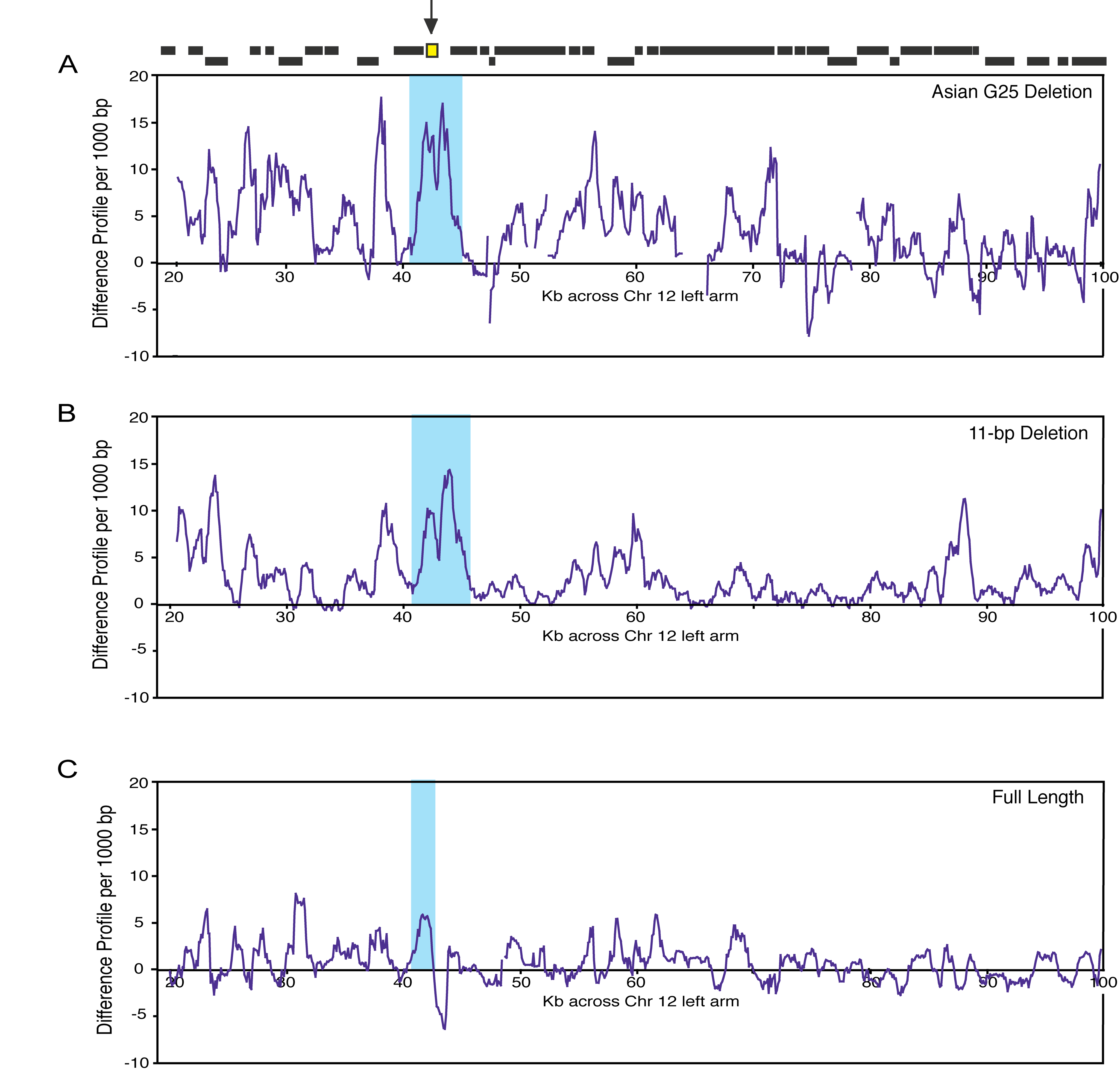

Supplement: Figure S2 — Difference profiles of between-group minus within-group variation. To identify regions with a skew in between-group and within-group variation, we calculated the difference profile as described in the text. Peaks where values were >1.5X the chromosome-wide average were identified and compared to peaks identified at the AQY2 locus. Blue windows highlight identified peaks over AQY2; ORF positions are shown above the figure as described in Figure 3. (1.54 MB TIF) [file pgen.1000893.s002.tif]

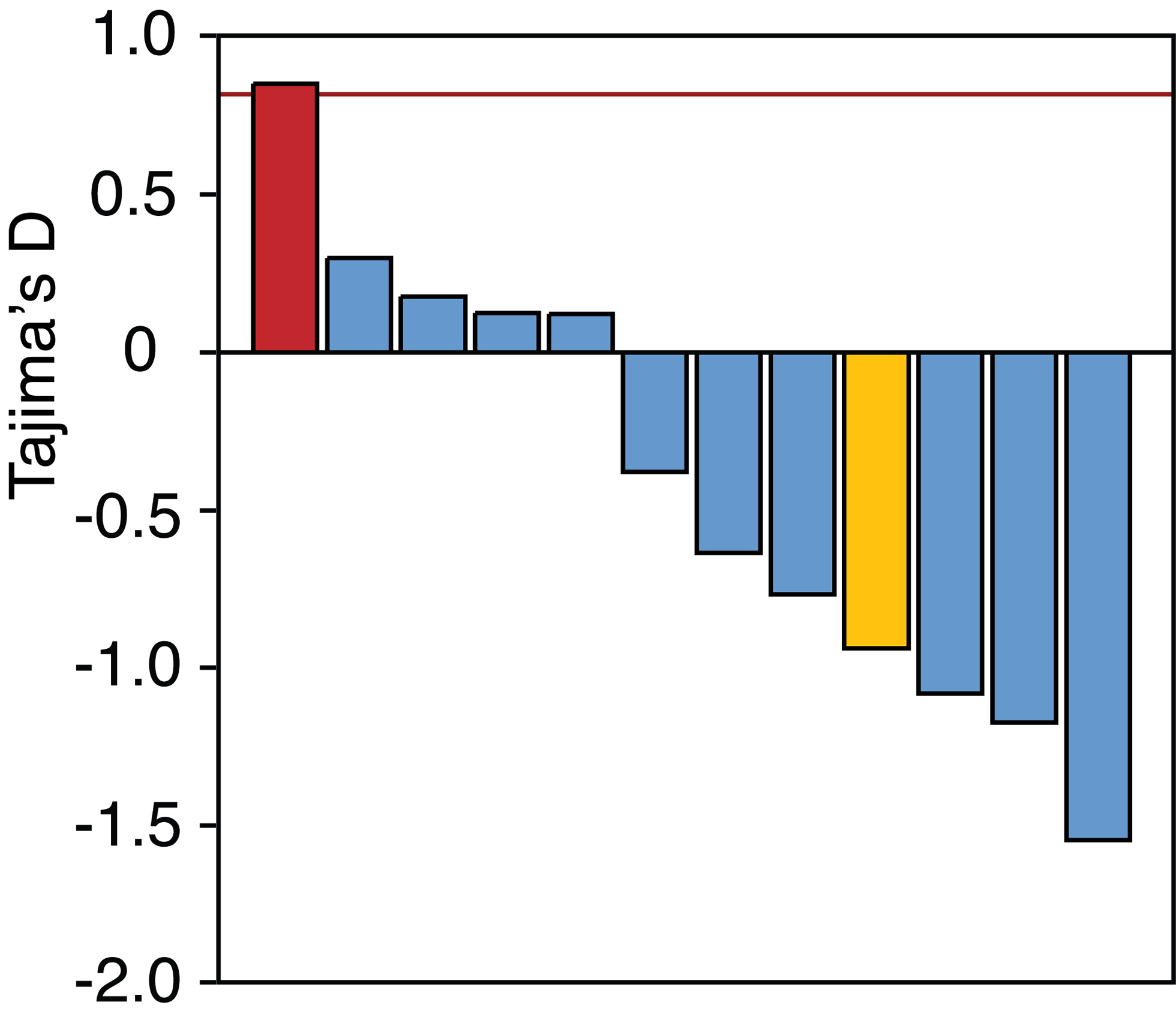

Supplement: Figure S3 — Tajima's D at AQY loci. Tajima's D was measured at 12 different loci with high-quality sequence data from [9],[20] and here, in 11 or 12 of 12 strains. Values for the AQY2 (red) and AQY1 (orange) coding sequences were compared to other intergenic loci. The 95% confidence interval (mean of non-AQY loci plus two standard deviations) is shown with a horizontal red line. Many genes show negative D values, consistent with previous genome wide estimates for S. cerevisiae [29]. (0.22 MB TIF) [file pgen.1000893.s003.tif]

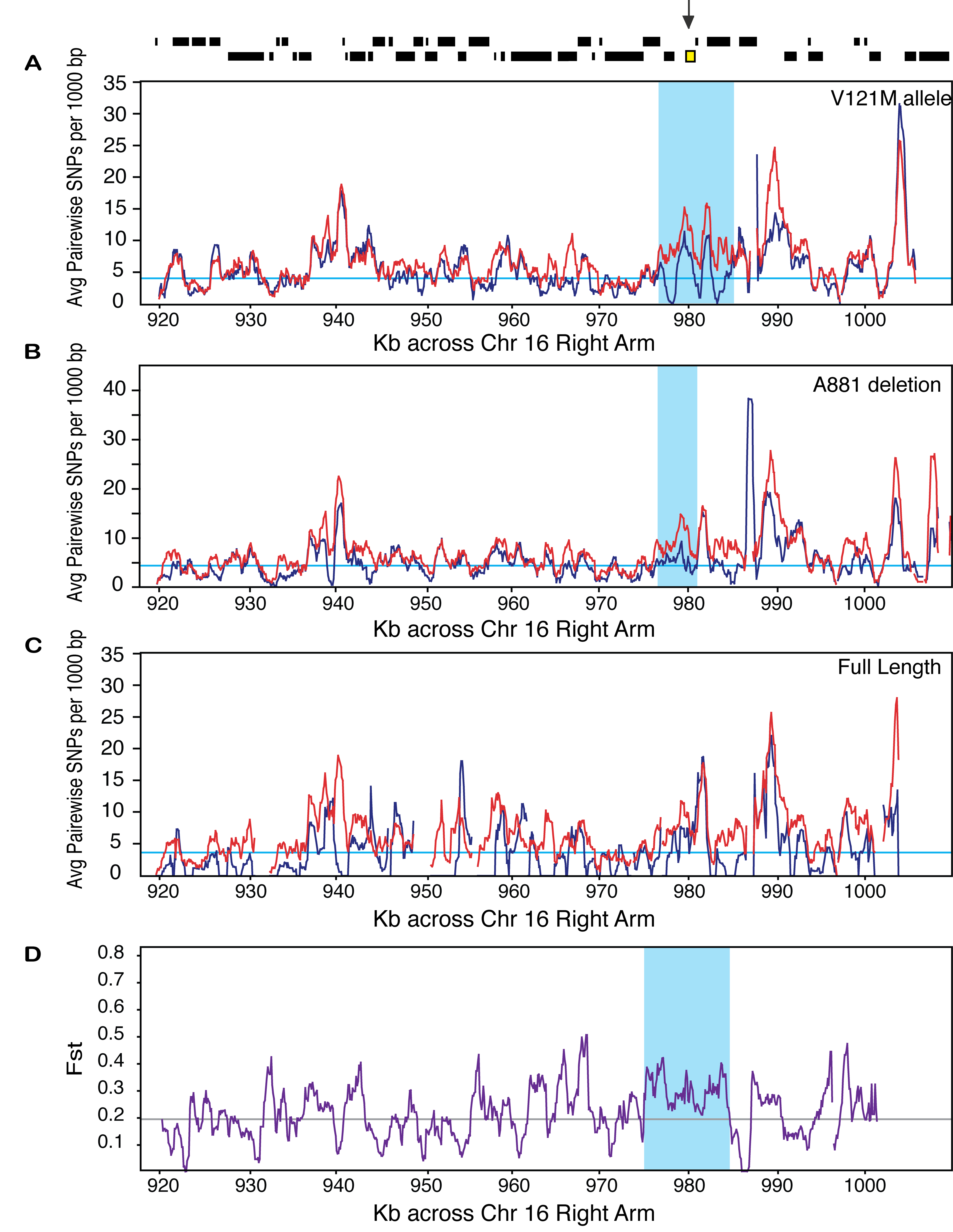

Supplement: Figure S4 — Within-group versus between-group variation at AQY1. As shown in Figure 3 for strains harboring the V121M allele, A881 deletion, or full-length AQY1. (3.16 MB TIF) [file pgen.1000893.s004.tif]

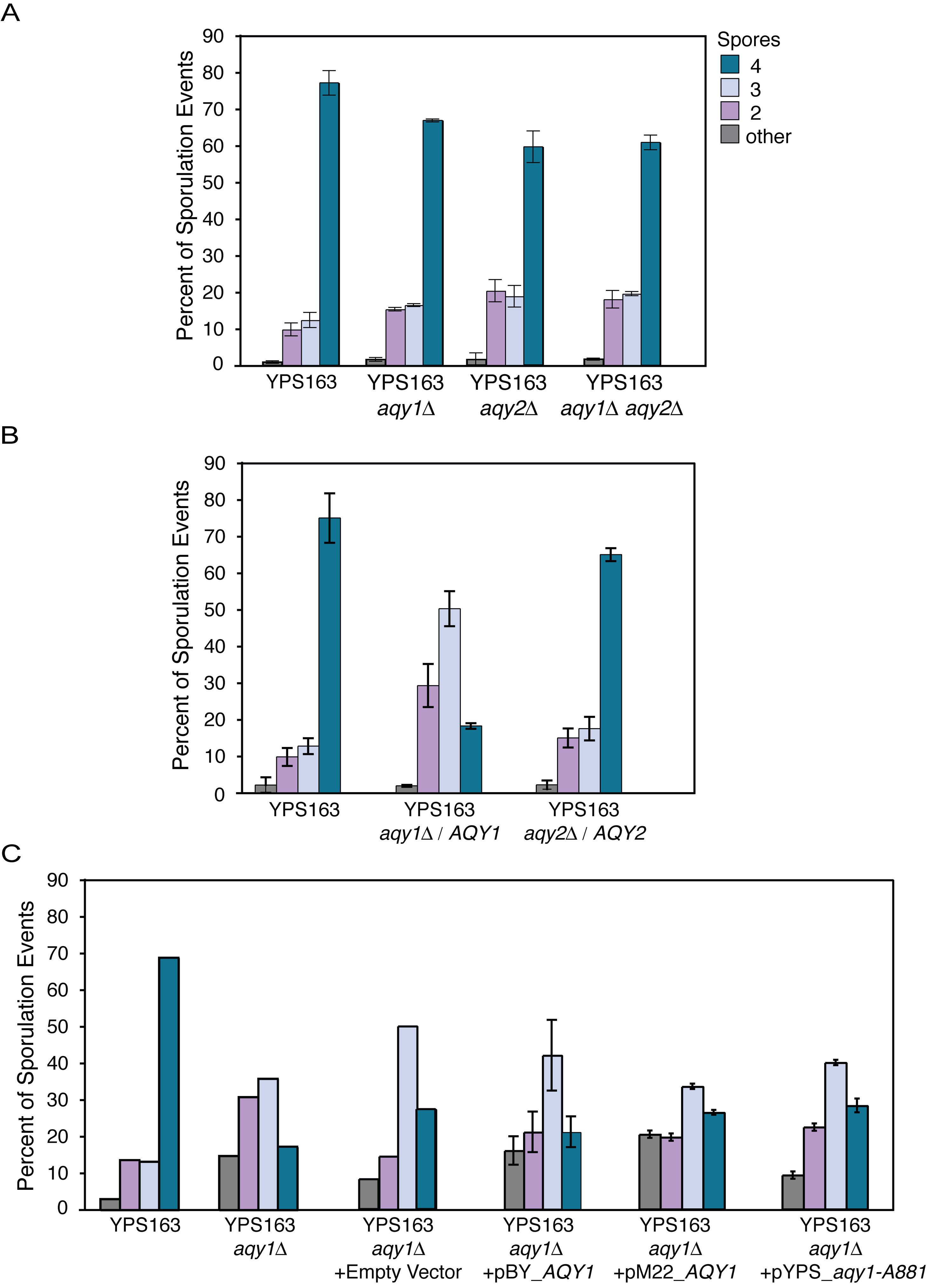

Supplement: Figure S5 — Sporulation defects in mutant strains. (A) Sporulation efficiency as shown in Figure 5 but measured at 9 days. (B) Haploinsufficiency is seen for heterozygous YPS163 AQY1/aqy1Δ but not YPS163 AQY2/aqy2Δ, suggesting AQY1 plays a more significant role in YPS163 sporulation. (C) Complementation experiments show that the YPS163 aqy1Δ sporulation defect is not complemented by AQY1 ORFs from S288c derivative BY4741 (BY), M22, or the YPS163 coding sequence with the A881 deletion. To avoid defects due to regulatory differences, each ORF was cloned between the 947-bp upstream and 747-bp downstream sequences from YPS163, exactly as for the pYPS_AQY1 clone that was able to complement the sporulation defect. Further confirming that these alleles are non-functional in our context, we found that none of these AQY1 versions contributed FT tolerance to BY4741 (data not shown), unlike the AQY1 allele from YPS163 (Figure 1C). Although the A881 allele of Aqy1 has been shown to produce a functional water transporter in an in vitro system, it was also shown to dramatically reduce protein levels [15], which may explain why it is not relevant in our in vivo analysis. (0.97 MB TIF) [file pgen.1000893.s005.tif]
