# Supplementary material for: Incipient Balancing Selection through Adaptive Loss of Aquaporins in Natural Saccharomyces cerevisiae Populations
Source: PLoS Genet. 2010 Apr 1;6(4):e1000893. doi: 10.1371/journal.pgen.1000893 (PMC2848549; doi:10.1371/journal.pgen.1000893)
Supplement: Table S3 — McDonald-Kreitman tables and p-values. McDonald-Kreitman tests were performed using DNASP on high-quality sequence data from this study, using S. paradoxus strain Q69.8, which had the best high-quality sequence coverage [21], as the outgroup. P-values were estimated using Fisher's exact test in DNASP 5.0 [45]. Significant tests are indicated with an asterisk. (0.06 MB DOC) [file pgen.1000893.s008.doc]

Table S3: MK tables and p-values

|  | **Polymorphism** | |  | **Fixed Differences** | |  |
| --- | --- | --- | --- | --- | --- | --- |
| ***AQY2: strains analyzed*** | **A** | **S** |  | **A** | **S** | **p-value** |
| 27 *S. cerevisiae* strains | 8 | 20 |  | 3 | 40 | 0.019 * |
| 27 *S. cerevisiae* strains (count deletions instead of A) | 3 | 20 |  | 0 | 40 | 0.045* |
| 10 strains: Full-length AQY2 allele | 2 | 7 |  | 3 | 55 | 0.12 |
| 6 strains: 11-bp deletion | 0 | 2 |  | 5 | 52 | 1 |
| 6 strains: Asian-1bp deletion | 2 | 2 |  | 4 | 49 | 0.051 |
|  |  |  |  |  |  |  |
| ***AQY1: strains analyzed*** |  |  |  |  |  |  |
| 25 *S. cerevisiae* strains (excluding Malasian strains with different Nt) | 5 | 4 |  | 11 | 49 | 0.026* |
| 9 strains: M121V mutation | 4 | 3 |  | 11 | 50 | 0.031* |
| 6 strains: Full-length AQY2 allele | 0 | 3 |  | 14 | 65 | 1 |
| 5 strains: long-tail | 3 | 4 |  | 14 | 65 | 0.14 |

MK tests were performed using DNASP on high-quality sequence data from this study, using *S. paradoxus* strain Q69.8, which had the best high-quality sequence coverage (Liti et al. 2009), as the outgroup. p-values estimated using Fisher’s exact test in DNASP 5.0 (Librado and Rozas 2009). Significant tests are indicated with an asterisk.
