# Supplementary material for: Incipient Balancing Selection through Adaptive Loss of Aquaporins in Natural Saccharomyces cerevisiae Populations
Source: PLoS Genet. 2010 Apr 1;6(4):e1000893. doi: 10.1371/journal.pgen.1000893 (PMC2848549; doi:10.1371/journal.pgen.1000893)
Supplement: Table S4 — Input data for ML-HKA test. a Sample size (number of strains), b number of segregating sites, c number of divergent sites. S. paradoxus strain Q69.8 was used as the outgroup [21]. We applied the multi-locus HKA method of Wright and Charlesworth [23] to test for selection at AQY2 and AQY1, compared to 7 intergenic sequences with data for both S. cerevisiae and S. paradoxus [9]. Each fragment is denoted by the chromosome and start position on that chromosome. Due to a large deletion removing the front part of the gene in Malaysian strains, the Malaysian AQY1 allele started at position 100, removing of an upstream, inframe ATG and 30 additional basepairs that were clearly not orthologous to the full-length AQY1 from other strains. Intergenic regions were analyzed after removing two clearly non-orthologous regions from all strains (350 bp from the chr2 fragment and 387 bp from the chr16 fragment), the result of apparent recombination in subgroups of strains. This dataset amounted to 183 and 150 silent positions in AQY2 and AQY1, respectively, and 3,337 scorable sites across 7 intergenic fragments. (0.07 MB DOC) [file pgen.1000893.s009.doc]

Table S4: Input data for MLHKA

| Locus Name | Number Silent Sites Assessed | N^a^ | S^b^ | D^c^ | Theta |
| --- | --- | --- | --- | --- | --- |
| *AQY2* | 183 | 27 | 20 | 40 | 0.02827 |
| *AQY1* | 150 | 28 | 5 | 41 | 0.00857 |
| chr2_389212 | 344 | 42 | 17 | 96 | 0.01148 |
| chr4_1166227 | 776 | 41 | 29 | 158 | 0.00873 |
| chr5_311196 | 585 | 42 | 14 | 60 | 0.00505 |
| chr6_113980 | 648 | 39 | 30 | 151 | 0.01132 |
| chr9_30049 | 390 | 38 | 23 | 134 | 0.01404 |
| chr10_96528 | 420 | 34 | 34 | 115 | 0.01572 |
| chr16_926934 | 174 | 37 | 11 | 29 | 0.01514 |

^a^ Sample size (number of strains), ^b^ number of segregating sites, ^c^ number of divergent sites. *S. paradoxus* strain Q69.8 was used as the outgroup (Liti et al. 2009). We applied the multi-locus HKA method of Wright and Charlesworth (Wright and Charlesworth 2004) to test for selection at *AQY2* and *AQY1*, compared to 7 intergenic sequences with data for both *S. cerevisiae* and *S. paradoxus* (Kvitek et al. 2008). Each fragment is denoted by the chromosome and start position on that chromosome. Due to a large deletion removing the front part of the gene in Malaysian strains, the Malaysian *AQY1* allele started at position 100, removing of an upstream, inframe ATG and 30 additional basepairs that were clearly not orthologous to the full-length *AQY1* from other strains. Intergenic regions were analyzed after removing two clearly non-orthologous regions from all strains (350bp from the chr2 fragment and 387bp from the chr16 fragment), the result of apparent recombination in subgroups of strains. This dataset amounted to 183 and 150 silent positions in *AQY2* and *AQY1*, respectively, and 3,337 scorable sites across 7 intergenic fragments.
