# Supplementary material for: Incipient Balancing Selection through Adaptive Loss of Aquaporins in Natural Saccharomyces cerevisiae Populations
Source: PLoS Genet. 2010 Apr 1;6(4):e1000893. doi: 10.1371/journal.pgen.1000893 (PMC2848549; doi:10.1371/journal.pgen.1000893)
Supplement: Table S5 — Results of ML-HKA analysis. ML-HKA tests were run under a model in which all loci were evolving neutrally and compared to models in which AQY2, AQY1, or AQY2 and AQY1 were under selection. The program was run with chain length 100,000 in all cases. P-values were calculated based on the chiX distribution of the likelihood statistic (2*(ln Lselection model–ln Lneutral model) listed degrees of freedom (df), as described (Wright and Charlesworth 2004). There was no increase in significance when all sites in AQY2 and AQY1 were treated as silent (justified since the genes are non-functional) (data not shown). We also ran the analysis separately for only strains in Full-length group, Asian G25 deletion group, or 11 bp-deletion group. Variation at AQY2 and AQY1 within each group was compared to variation at other loci only for strains in that group. The results were the same when the genes in question were scored against intergenic sequences from all available strains. None of the tests were significant in any case. (0.06 MB DOC) [file pgen.1000893.s010.doc]

**Table S5: Results of ML-HKA analysis**

| Model | Description | ln Likelihood | Model Comparison | Likelihood Statistic (df) | p-value |
| --- | --- | --- | --- | --- | --- |
| A | All sites neutral | -58.7097 |  |  |  |
| B | *AQY2* under selection | -56.2833 | B vs A | 4.851 (1) | 0.028* |
| C | *AQY1* under selection | -58.2702 | C vs A | 0.879 (1) | 0.348 |
| D | AQY2 + AQY1 selection | -56.0142 | D vs A | 5.381 (2) | 0.02* |
|  |  |  | D vs B | 0.538 (1) | 0.463 |

MLHKA was run under a model in which all loci were evolving neutrally compared to models in which *AQY2*, *AQY1*, or *AQY2* and *AQY1* were under selection. The program was run with chain length 100,000 in all cases. P-values were calculated based on the chiX distribution of the likelihood statistic (2*(ln L_selection model_ – ln L_neutral model_) listed degrees of freedom (df), as described (Wright and Charlesworth 2004).

There was no increase in significance when all sites in *AQY2* and *AQY1* were treated as silent (justified since the genes are non-functional) (data not shown). We also ran the analysis separately for only strains in Full-length group, Asian G25 deletion group, or 11bp-deletion group. Variation at *AQY2* and *AQY1* within each group was compared to variation at other loci only for strains in that group. The results were the same when the genes in question were scored against intergenic sequences from all available strains. None of the tests were significant in any case.
